# Thymic stromal lymphopoietin-induced HOTAIR activation promotes endothelial cell proliferation and migration in atherosclerosis

**DOI:** 10.1042/BSR20170351

**Published:** 2017-07-12

**Authors:** Yudong Peng, Kai Meng, Lili Jiang, Yucheng Zhong, Yong Yang, Yin Lan, Qiutang Zeng, Longxian Cheng

**Affiliations:** Department of Cardiology, Union Hospital, Tongji Medical College, Huazhong University of Science and Technology, Wuhan city, Hubei province, China

**Keywords:** atherosclerosis, endothelial cell, HOTAIR, ox-LDL, TSLP

## Abstract

Endothelial cells’ (EC) injury is a major step for the pathological progression of atherosclerosis. Recent study demonstrated that thymic stromal lymphopoietin (TSLP) exerts a protective role in atherosclerosis. However, the effect of TSLP and the exact molecular mechanism involved in EC remains unknown. In the present study, we found that long noncoding RNA (lncRNA) HOTAIR was much lower in EC from atherosclerotic plaque. Functional assays showed that HOTAIR facilitated cell proliferation and migration, and suppressed apoptosis in EC. Moreover, we demonstrated that TSLP functions upstream of HOTAIR. We found that serum level of TSLP was decreased in atherosclerosis patients and serum TSLP level positively correlated with HOTAIR expression in EC. Further investigation demonstrated that TSLP activated HOTAIR transcription through PI3K/AKT-IRF1 pathway and then regulates the EC proliferation and migration. TSLP-HOTAIR axis also plays a protective role in low-density lipoprotein (ox-LDL)-induced EC injury. Taken together, TSLP-HOTAIR may be a potential therapy for EC dysfunction in atherosclerosis.

## Introduction

Atherosclerosis is a chronic inflammatory disease characterized by the accumulation of lipids and fibrous elements in the arterial wall of large- and medium-sized arteries, and is the main cause of heart disease and stroke [1,2]. Endothelial cells (EC) injury is a major step for the pathological progression of atherosclerosis. When exposed to various insults, such as oxidative stress, ECs are activated and express adhesion molecules, gradually resulting in certain pathological changes, including apoptosis and proliferation inhibition [3,4]. Therefore, to understand the underlying molecular mechanism involved in EC injury is of great importance for atherosclerosis prevention.

Thymic stromal lymphopoietin (TSLP), which belongs to an IL-7 like cytokine family, is mainly expressed by epithelial cells. Previous studies have reported that TSLP signal was activated through a heterodimeric receptor complex consisting of an IL-7 receptor-α chain and a unique TSLP receptor (TSLPR). TSLPs are often activated by infection related agents, allergens and cytokines including IL-4, IL-1 and TNF-α [5]. After stimulation of TSLPR induced by these factors, the receptor complex transmits the signal by activating PI3K/AKT pathway [6–8]. A recent study demonstrated that apolipoprotein E-deficient (ApoE^−/−^) mice treated with TSLP developed significantly fewer atherosclerotic plaques in the aortic root compared with controls, along with decreased inflammation in the aorta, indicating a protective role of TSLP in atherosclerosis [9]. However, the expression level of TSLP in atherosclerosis patients, the effect of TSLP on EC and the exact molecular mechanism remains unknown.

Recently, increasing evidence demonstrated that noncoding RNAs (ncRNAs) play important roles in the regulation of gene expression via multiple mechanisms [10]. Long noncoding RNAs (lncRNAs) are defined as transcripts that are longer than 200 nts without protein-coding function. Dysregulation of lncRNAs are closely associated with cardiovascular diseases. For example, lncRNA ANRIL expression level is associated with the risk for coronary atherosclerosis, carotid arteriosclerosis and peripheral artery disease [11–13]. HOTAIR is a 2.2-kb long lncRNA that is transcribed from antisense strand of *HOXC* gene cluster present in chromosome 12 [14]. HOTAIR is a key regulator of chromatin dynamics and is abnormally expressed in various carcinomas such as colorectal cancer, pancreatic cancer, hepatocellular carcinoma and renal cancer [15–18]. HOTAIR interacts with polycomb repressive complex 2 (PRC2) and is necessary for PRC2 occupancy and histone H3 lysine-27 trimethylation (H3K27me3) of target genes. HOTAIR is involved in several processes associated with tumorigenesis and tumor progress such as cell migration, proliferation, apoptosis and metastasis [19,20]. A recent study showed that AKT activation suppressed interferon regulatory factor (IRF1) expression and decreased the binding of IRF1 to the HOTAIR promoter, subsequently facilitated HOTAIR transcription [21]. However, the functional role of HOTAIR in EC of atherosclerosis and whether HOTAIR is regulated by TSLP-induced activation of AKT pathway remains unclear. In the present study, we analyzed the expression pattern of HOTAIR in EC of atherosclerosis and detected its effect on EC proliferation, apoptosis and migration. Moreover, we also determined the functional relationship of HOTAIR and TSLP signal.

## Materials and methods

### Tissue samples and EC isolation

The complete details of the entire study design and procedures involved were in accordance with the Declaration of Helsinki. Informed written consent was obtained from all the subjects. The study process was approved by the Ethics Review Committee of the Tongji Medical College. A total of 40 atherosclerotic patients were included in the present study after angiographic documentation of coronary artery disease (CAD). Atherosclerotic plaques were collected from patients undergoing vascular surgery or at autopsy. Primary EC were isolated as previously described [22]. The serum samples were obtained from these 40 atherosclerotic patients and other 40 healthy donors. The healthy donors did not suffer from any disease. The exclusion criteria were as follows: presenting with advanced liver disease, chronic-immune mediated diseases, renal failure, thromboembolism or infections during the last 4 weeks.

### ELISA for TSLP detection

Serum TSLP levels were determined by using an ELISA kit from eBioscience according to the manufacturer’s instructions.

### Cell culture

Two EC cell lines, HUVEC and HAEC were obtained from Cell Bank of Chinese Academy of Sciences. Cells were cultured at 37°C in an atmosphere containing 5% CO_2_ and in RPMI-1640 medium (Gibco) supplemented with 10% FBS (Gibco).

### RNA extraction and quantitative Real-time PCR (q-RT PCR)

Total RNA was isolated using TRIzol reagent (Invitrogen, Carlsbad, CA) according to the manufacturer’s instructions. cDNA was synthesized by using PrimeScript First Strand cDNA Synthesis Kit (Takara). GAPDH served as an endogenous control. Real-time PCR was performed on the Applied Biosystems 7300 Real-Time PCR system using SYBR Green dye (Applied Biosystems, Foster City, CA) as manufacturer’s instructions. All detections were performed in triplicate. The 2^−ΔΔ*C*^_^t^_method was applied to calculate relative transcript levels. The primer sequence for HOTAIR was provided as follows: F-AGACCCTCAGGTCCCTAATATC, R-CCCTACTGCAGGCTTCTAAATC.

### Western blot

Cells were lysed in RIPA lysis buffer with protease inhibitor cocktail (Roche). Lysis was subjected to SDS/PAGE and transferred on to a PVDF membrane (Millipore, Billerica, MA). The membranes were blocked with 5% non-fat dry milk at room temperature for 1 h and then incubated with anti-IRF1 (Abcam), AKT (Cell Signaling), P-AKT (Cell signaling) or GAPDH (Santa Cruz) antibodies for overnight at 4°C. p-Akt and total Akt were analyzed in different membranes. After washing, the membrane was reacted with HRP–conjugated secondary antibodies (Jackson) for 1 h at room temperature. Bands were visualized using the ECL detection system (GE Healthcare, Chalfont St Giles, U.K.).

### Transfection

HOTAIR was amplified from HUVEC and cloned into expression plasmid pCMV (Addgene). Cells were transfected with pCMV or pCMV-HOTAIR by using Turbofect (Thermo) following the manufacturer’s instructions. siRNAs targeting HOTAIR was purchased from Ribobio Company (Guangzhou, China). The target sequence for HOTAIR was as follows: si1, CTGGGAGGCGCTAATTAAT; si2, AGGCAAATGTCAGAGGGTT. Cells transfected with scramble siRNA is taken as control group. siRNAs were transfected into cells by using using Hiperfect (Qiagen) following the manufacturer’s instructions. After 48 h of transfection, the following experiments were performed.

### Treatment

A total of 48 h after transfection, the cells were treated with different concentrations of oxidized low-density lipoprotein (ox-LDL, Beijing Xiesheng BioTechnology Limited, Beijing, China) for 12 h or with 40 μg/ml ox-LDL for different time points. Cells were treated with recombinant human TSLP (rhTSLP) (R&D Systems) at different concentration for 24 h, with vehicle as control. We treated HUVEC with rhTSLP (10 pg/ml) and TSLP (5 μg/ml, R&D Systems) or TSLPR antibody (5 μg/ml, R&D Systems) or PI3K inhibitor LY294002 (20 μmol/l, Selleck) for 24 h. After drug treatment, the cells were used for flow cytometry or RNA extraction or cell viability detection.

### Migration assay

A total of 1 × 10^5^ cells were seeded in serum-free RPMI-1640 in the upper chamber of an 8-μm pore 24-well transwell insert. The lower chamber was filled with RPMI-1640 containing 10% FBS. The 24-well plates were then incubated for 24 h in an atmosphere at 37°C. Cells that migrated through the membranes were fixed in 4% paraformaldehyde and then stained by Crystal Violet. Cells were counted in ten random fields under a microscope.

### CCK-8 assay

Cell proliferation and viability was determined by the CCK-8 (Dojindo) according to the manufacturer’s instructions. Cell proliferation rate was detected by measuring the absorbance at 450 nm with the microplate reader (Bio–Rad). The OD value of each group at day 1 was taken as control.

### Apoptosis assay

The apoptosis was detected by using Apoptosis Detection Kit (KeyGEN, Nanjing, China) according to the manufacturer’s instructions. Cells were stained with fluorescein isothiocyanate–conjugated Annexin V and 7-Aminoactinomycin D (7-AAD). Cells were then analyzed with a FACScan flow cytometer and the data were analyzed by Kaluza software. Annexin V labels externalized phosphatidylserine and 7-ADD labels DNA.

### ChIP

ChIP assay was performed by using the EZ-Magna Chromatin Immunoprecipitation Kit (Millipore) according to the manufacturer’s protocol. Immunoprecipitate complexes were cultured with an anti-IRF1 antibody (Abcam) or negative control IgG antibody (Millipore) overnight at 4°C. The captured DNA was used for quantative-PCR (qPCR) analysis. The primers used for HOTAIR promoter sequence were provided as follows: F-CATGCTGTCTTAGCGGATCTT, R-ATGTCTTGCTGCTCGGATTAG.

### Statistical analysis

All statistical analysis was performed using SPSS 17.0 software. All results were expressed as mean ± S.D. and were statistically evaluated using a one-way ANOVA followed by the *t* test. For all tests, *P*<0.05 was taken as statistically significant.

## Results

### HOTAIR promotes EC proliferation and migration

**Figure 1:**
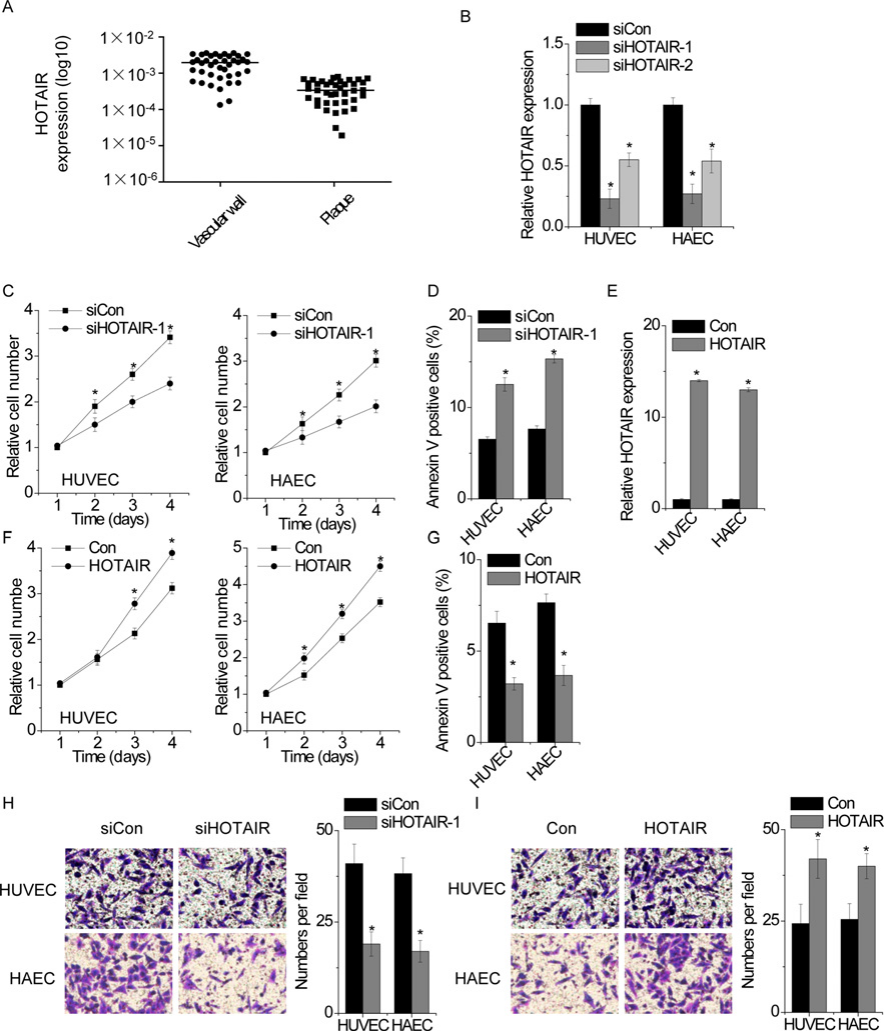
HOTAIR promotes EC proliferation and migration in EC (**A**) The expression of HOTAIR in 40 pairs of EC from atherosclerotic plaques and the corresponding vascular wall were detected by qPCR. (**B**) Two different siRNAs targeting HOTAIR were transfected into HUVEC and HAEC. The relative level of HOTAIR in control and HOTAIR-silenced cells was detected by qPCR (one-way ANOVA followed by the *t* test). (**C**) The relative cell proliferation rate was determined by CCK-8 assay in control or HOTAIR-silenced cells. The OD value of each group in day 1 was taken as control. (**D**) Cells with or without HOTAIR silence were stained with a combination of Annexin V and 7-AAD and analyzed by FACS. Cells positive for annexin V staining were counted as apoptotic cells. (**E**) HUVEC and HAEC were transfected with empty vector or HOTAIR. The HOTAIR expression in control and HOTAIR-overexpressed cells was analyzed by qPCR. (**F**) The growth rate of control and HOTAIR-overexpressed cells were detected by CCK-8 assay. (**G**) Cells with or without HOTAIR overexpression were stained with a combination of Annexin V and 7-AAD and analyzed by FACS. Cells positive for Annexin V staining were counted as apoptotic cells. (**H**) The migration of control and HOTAIR-silenced cells were determined by transwell assay. (**I**) The migration of control and HOTAIR-overexpressed cells were determined by transwell assay. All experiments were repeated three times. Data are shown as mean ± S.D. *P<0.05. The difference between the two groups was statistically evaluated using Student’s *t* test.

First, we examined the expression pattern of HOTAIR in EC isolated from atherosclerotic plaque in atherosclerosis patients. We detected HOTAIR expression in 40 pairs of EC isolated from atherosclerotic plaques and the corresponding vascular wall by qPCR. We found that HOTAIR expression was significantly lower in EC from atherosclerotic plaque compared with corresponding vascular wall (Figure 1A).

Next, we determined the functional role of HOTAIR in EC by gain- and loss-of-function assays in two EC lines, HUVEC and HAEC. Cells were transfected with two different siRNAs against HOTAIR. Both the siRNAs significantly suppressed HOTAIR expression, and the target site one (siHOTAIR-1) is the most effective site that is chosen for the following experiments (Figure 1B). CCK-8 assay showed that knockdown of HOTAIR inhibited the proliferation of both HUVEC and HAEC compared with cells transfected with non-targeting siRNA (Figure 1C). In addition, we performed the FACS to analysis the effect of HOTAIR on cell apoptosis. We found that knockdown of HOTAIR significantly promoted EC apoptosis (Figure 1D). In contrast, we transfected HOTAIR into HUVEC and HAEC (Figure 1E). Up-regulation of HOTAIR markedly enhanced proliferative ability and decreased cell apoptosis (Figure 1F, G).

Migration of EC has also been implicated to be critical in the progression of atherosclerotic lesions [23,24]. We suspected that HOTAIR may play a role in EC migration. We performed transwell assay to detect the migration mediated by HOTAIR. We found that depletion of HOTAIR significantly suppressed cell migration in HUVEC and HAEC (Figure 1H), whereas overexpression of HOTAIR showed the opposite effect (Figure 1I).

### HOTAIR is up-regulated by TSLP through AKT pathway

**Figure 2:**
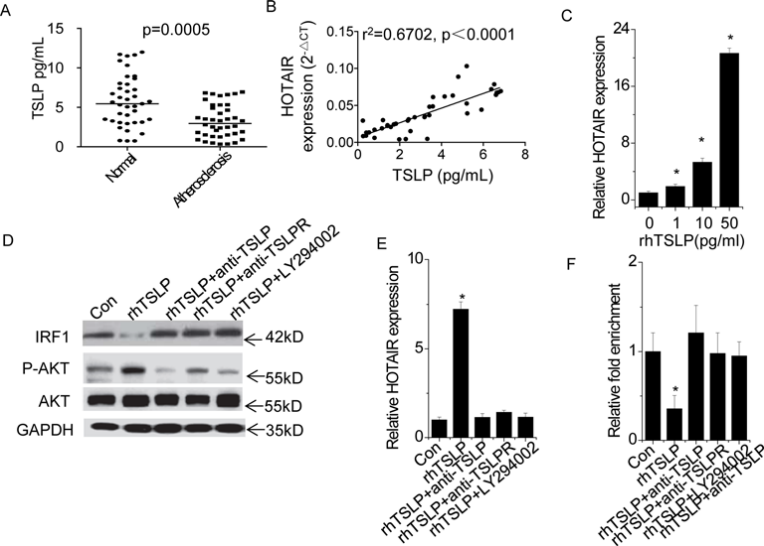
HOTAIR is up-regulated by TSLP through AKT pathway (**A**) The TSLP level in serum was detected in 40 atherosclerotic patients and 40 healthy donors by ELISA assay (Student’s *t* test). (**B**) The correlation analysis between serum TSLP level and HOTAIR expression of EC in atherosclerotic patients (Pearson’s correlation analysis). (**C**) HUVEC were treated with different concentration of rhTSLP for 24 h. The vehicle is PBS, and the expression change of HOTAIR was determined by qPCR. (**D**) HUVEC were treated with 10 ng/ml rhTSLP and and TSLP (5 μg/ml) or TSLPR antibody (5 μg/ml) or PI3K inhibitor LY294002 (20 μmol/l) for 24 h. And the phosphorylation of AKT and IRF1 level was analyzed by western blot. (**E**) HUVEC were treated with 10 ng/ml rhTSLP and and TSLP (5 μg/ml) or TSLPR antibody (5 ug/ml) or PI3K inhibitor LY294002 (20 μmol/l) for 24 h. And the expression change of HOTAIR was determined by qPCR. (**F**) HUVEC were treated with 10 ng/ml rhTSLP and and TSLP (5 ug/ml) or TSLPR antibody (5 ug/ml) or PI3K inhibitor LY294002 (20 μmol/l) for 24 h. We performed anti-IRF1 ChIP to analyze the binding level of IRF1 on HOTAIR promoter region. All experiments were repeated three times. Data are shown as mean ± S.D. *P<0.05. The difference between different groups was statistically evaluated using one-way ANOVA followed by the *t* test.

Next, we determined the relationship between TSLP signal and HOTAIR. We detected the serum TSLP levels in 40 atherosclerotic patients and 40 healthy donors by ELISA assay. Serum TSLP levels were much lower in atherosclerosis patients than in healthy donors (Figure 2A). In addition, we analyzed the correlation between serum TSLP level and HOTAIR expression of EC in atherosclerosis patients. As shown in Figure 2B, a positive correlation between serum TSLP level and HOTAIR expression of EC in atherosclerosis patients was observed (r^2^ =0.6702, *P*<0.0001), indicating that HOTAIR expression may be regulated by TSLP.

To clarify the influence of TSLP on HOTAIR expression, we treated HUVEC with different concentrations of rhTSLP. The results showed that TSLP significantly increased HOTAIR expression in a dose-dependent manner (Figure 2C). We suspected that whether up-regulation of HOTAIR was induced by TSLP treatment via TSLP-TSLPR-PI3K/AKT pathway. We treated HUVEC with rhTSLP (10 pg/ml) and TSLP (5 μg/ml) or TSLPR antibody (5 μg/ml) or PI3K inhibitor LY294002 (20 μmol/l). We found that phosphorylation level of AKT was increased by rhTSLP, which was rescued by TSLP- or TSLPR-neutralizing antibody or LY294002 treatment (Figure 2D). Consistently, TSLP- or TSLPR-neutralizing antibody or LY294002 treatment abolished the up-regulation of HOTAIR induced by rhTSLP (Figure 2E). However, inhibition of IL-7 receptor-α by neutralizing antibody did not influence TSLP-induced HOTAIR activation (Supplementary Figure S1). Our results indicated that TSLP activates HOTAIR expression through TSLR-PI3K/AKT pathway.

Previous study demonstrated that osteopontin (OPN) regulated PI3K/AKT activation and IRF1 expression, subsequently influencing the expression of HOTAIR [25]. Similarly, we found that IRF1 expression was significantly decreased by TSLP and rescued by TSLP or TSLPR antibody or LY294002 treatment (Figure 2D). Furthermore, we performed a ChIP assay followed by qPCR to detect the binding level of IRF1 at *HOTAIR* promoter region. Inhibition of TSLP-TSLPR-PI3K/AKT-IRF1 pathway significantly abolished the rhTSLP-induced decrease in the IRF1 occupancy at *HOTAIR* promoter (Figure 2F). Taken together, these data indicated that TSLP bind to its receptor TSLPR, activates PI3K/AKT pathway to suppress IRF1 expression and subsequently stimulate the expression of HOTAIR.

### HOTAIR is crucial for the effect of TSLP on EC

**Figure 3:**
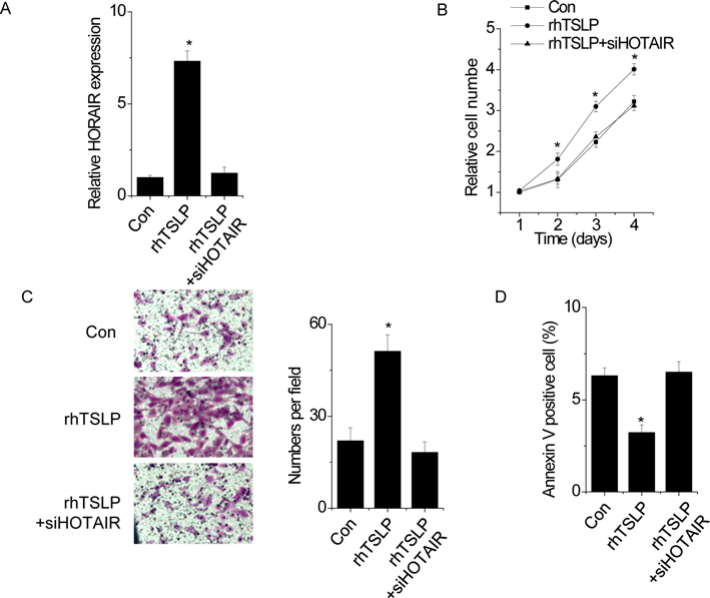
HOTAIR is crucial for the effect of TSLP on EC (**A**) HUVEC were treated with or without rhTSLP and transfected with siRNA targeting HOTAIR. The HOTAIR expression was detected by qPCR. (**B**) The cell proliferation was determined by CCK-8. Silence of HOTAIR abolished the promotion of rhTSLP on HUVEC proliferation (Student’s *t* test). (**C**) The cell migration assay was performed to determine the effect of HOTAIR knockdown on rhTSLP-induced migration in HUVEC. (**D**) The cell apoptosis was detected by FACS. Silence of HOTAIR abolished the inhibition of rhTSLP in HUVEC apoptosis. All experiments were repeated three times. Data are shown as mean ± S.D. *P<0.05. The difference between different groups was statistically evaluated using one-way ANOVA followed by the *t* test.

We next determined the significance of HOTAIR in TSLP-mediated phenotypes of EC. We transfected HOTAIR siRNA into rhTSLP-treated HUVEC (Figure 3A). Interestingly, we found that rhTSLP stimulated proliferation (Figure 3B) and migration (Figure 3C), and inhibited cell apoptosis in HUVEC (Figure 3D). Depletion of HOTAIR almost abolished these effects, indicating that the cellular proliferation, apoptosis and migration of EC mediated by TSLP/TSLPR pathway is dependent on HOTAIR.

### TSLP-HOTAIR axis protects EC from injury caused by ox-LDL

**Figure 4:**
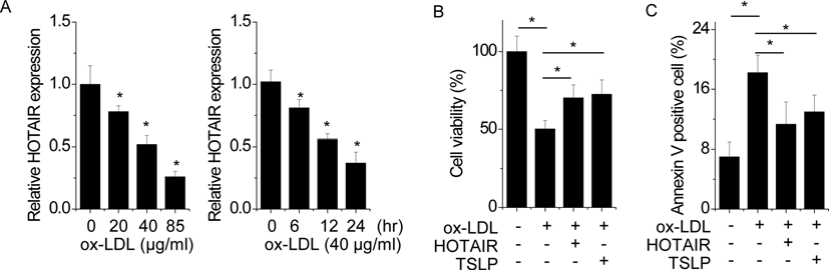
TSLP-HOTAIR axis protects EC from injury caused by ox-LDL (**A**) The HUVEC were treated with different concentration of ox-LDL for 12 h (left) or 40 μg/ml ox-LDL for different time points (right). The HOTAIR expression change was determined by qPCR. (**B**) The HUVEC were pretreated with TSLP (5 ug/ml) 40 μg/ml for 24 h or pretransfected with HOTAIR, and then treated with or without 40 μg/ml ox-LDL for 24 h. The cell viability was detected by CCK-8 assay. (**C**) The HUVEC were pre-treated with TSLP (5 ug/ml) 40 μg/ml for 24 h or pre-transfected with HOTAIR, and then treated with or without 40 μg/ml ox-LDL for 24 h. The cell apoptosis was analyzed by FACS. All experiments were repeated three times. Data are shown as mean ± S.D. *P<0.05. The difference between different groups was statistically evaluated using one-way ANOVA followed by the *t* test.

It has been reported that ox-LDL-induced oxidative injury is a critical step in EC injury and atherosclerotic plaque formation [26]. We speculated that TSLP-HOTAIR axis may also play a role in ox-LDL-induced EC damage. Firstly, we detected whether ox-LDL could influence the HOTAIR expression in EC. HUVEC were exposed to different concentrations and time point of ox-LDL. We found that HOTAIR expression was significantly decreased in a time- and dose-dependent manner (Figure 4A). To investigate the effect of TSLP-HOTAIR in EC, we measured the cell viability by CCK-8 assay. HUVECs explored to ox-LDL showed significantly decreased cell viability, while pretreated with TSLP or pretransfected with HOTAIR markedly ameliorated cell injury (Figure 4B). Furthermore, cell apoptosis was detected by FACS. The results showed that ox-LDL-treated cells showed higher percentage of positive Annexin V, whereas pretreated with TSLP or pretransfected with HOTAIR dramatically decreased ox-LDL-induced cell apoptosis (Figure 4C). Taken together, our findings suggest that TSLP-HOTAIR exerts a protective function in ox-LDL-mediated EC injury.

## Discussion

In the present study, HOTAIR expression was found to be much lower in EC from atherosclerotic plaques. Given that lncRNAs are critical for gene regulatory networks, our present study focused on the potential functional role regulated by HOTAIR. Our data demonstrated that HOTAIR facilitated cell proliferation and migration, and suppressed apoptosis in EC. Endothelial dysfunction is one of the initial steps in the development of atherosclerosis. EC showed a decreased proliferation and migration and increased apoptosis [3]. Our results suggest that HOTAIR play a protective role for EC injury. Next, we gain insight into the upstream of HOTAIR. We found that TSLP activated HOTAIR transcription through PI3K/AKT-IRF1 pathway, and then regulated the proliferative and migratory phenotypes of EC. In addition, we observed that serum TSLP level was much lower in atherosclerosis and there existed a positive correlation between TSLP and HOTAIR expression. Furthermore, TSLP-HOTAIR axis played a protective role in low-density lipoprotein (LDL)-induced EC injury. Taken together, TSLP-HOTAIR may be a potential therapy for EC dysfunction in atherosclerosis.

In atherosclerosis, EC activation triggers platelet aggregation, immunocytes adhesion and vascular smooth muscle cells proliferation. The proliferation and migration of EC have been implicated to be a critical step in the progression of atherosclerotic lesions [23,27]. TSLP has been shown to attenuate atherosclerosis in ApoE^−/−^ mice. TSLP administration increased the levels of anti-ox-LDL IgM and IgG1, but decreased the levels of IgG2a in plasma, along with increased numbers of CD4^+^LAP^+^ regulatory T cells (Tregs) in the spleen and decreased inflammation in the aorta [9]. However, the biological significance of TSLP in EC is still unknown in atherosclerosis. In the present study, for the first time, we found that serum TSLP level was much lower in atherosclerosis patients. TSLP promoted proliferation and migration, and suppressed apoptosis in EC, indicating that TSLP may attenuate atherosclerosis through regulation of EC besides immunity regulation. Mechanistic investigation revealed that TSLP activated PI3K/AKT pathway and inhibited IRF1 expression. Down-regulation of IRF1 subsequently increased the HOTAIR expression. Further rescue experiments demonstrated that TSLP modulated cell proliferation, apoptosis and migration in a HOTAIR-dependent manner. Furthermore, we demonstrated a positive correlation between serum TSLP level and HOTAIR expression of EC. Our results strongly indicated a close relationship between TSLP-HOTAIR pathway and atherosclerosis.

Ox-LDL-induced EC injury, which mimics oxidative injury to EC, is taken as an initial and critical step in the atherosclerotic process [26]. In the present study, for the first time, our results showed that HOTAIR could be suppressed by ox-LDL treatment in a dose- and time-dependent manner. Overexpression of HOTAIR or TSLP treatment significantly attenuated the suppression of cell viability and enhancement of cell apoptosis caused by ox-LDL. Our findings suggest that TSLP-HOTAIR axis exerts a protective function in ox-LDL-induced EC injury.

In conclusion, these results demonstrated that TSLP-PI3K/AKT-HOTAIR pathway positively regulates proliferation and migration of EC. These findings may reveal important insights into the role of lncRNAs in the pathogenesis and progression of atherosclerosis. TSLP and HOTAIR may serve as a novel therapy for atherosclerosis. However, numbers of specific lncRNAs might be involved in atherosclerotic disease. Thus, investigation of other lncRNAs and their functional association with atherosclerosis is necessary to overcome CADs.

## Abbreviations

7-AAD: 7-aminoactinomycin D
AKT: serine/threonine kinase 1
ANRIL: antisense noncoding RNA in the INK4 locus
CAD: coronary artery disease
CCK-8: cell counting kit-8
EC: endothelial cell
GAPDH: glyceraldehyde-3-phosphate dehydrogenase
HAEC: human artificial episomal chromosome
H3K27me3: histone H3 lysine-27 trimethylation
HOTAIR: HOX transcript antisense RNA
HUVEC: human vascular endothelial cells
HRP: horseradish peroxidase
IL-7: interleukin-7
IRF 1: interferon regulatory factor
IRF1: interferon regulatory factor 1
lncRNA: long noncoding RNA
ncRNA: noncoding RNA
OD: optical density
ox-LDL: oxidized low-density lipoprotein
PCR2: polycomb repressive complex 2
pCMV: a plasmid name.
PI3K: phosphatidylinositol 3-kinase
rhTSLP: recombinant human TSLP
TSLP: thymic stromal lymphopoietin
TSLPR: thymic stromal lymphopoietin receptor
